# Relationship of hypoxia-inducible factor 1α and p21^WAF1/CIP1 ^expression to cell apoptosis and clinical outcome in patients with gastric cancer

**DOI:** 10.1186/1477-7819-4-94

**Published:** 2006-12-13

**Authors:** Ken Mizokami, Yoshihiro Kakeji, Shinya Oda, Yoshihiko Maehara

**Affiliations:** 1 Department of Surgery and Science, Graduate School of Medical Science, Kyushu University, Fukuoka, Japan

## Abstract

**Background:**

Hypoxia-inducible factor-1α (HIF-1α) plays an essential role in oxygen homeostasis. The expression of HIF-1α-inducible genes is associated with tumor progression. p21 mediates cell cycle arrest and is one of the downstream genes targeted by HIF-1.

**Patients and methods:**

We examined the relationship between HIF-1α and p21 expression, apoptosis and tumor progression using tissue specimens obtained surgically from 126 patients with gastric cancer.

**Results:**

Immunohistochemical analysis indicated that loss of p21 expression correlated positively with patient age and tumor size. Lymph node metastasis was significantly more frequent in tumors with loss of p21 expression (P = 0.022). HIF-1α-positive/p21-negative tumors had a lower apoptotic index than any other tumor samples, and patients with HIF-1α-positive/p21-negative tumors also had a significantly poorer prognosis than the other patient populations.

**Conclusion:**

These results suggest that loss of HIF-1α-dependent p21 expression results in decreased apoptosis, increased cell survival and more aggressive tumors.

## Background

The unregulated growth of cancer cells often results in hypoxic conditions in tumor cell masses. Tumor hypoxia results from an imbalance between elevated consumption of oxygen in the rapidly cycling tumor cells and insufficient oxygen supply due to the lack of a physiological vascular network. Multicellular organisms have evolved cellular mechanisms that mediate a cascade of adaptive molecular responses to hypoxia. HIF-1α is a transcription factor that activates gene expression by binding to the hypoxia responsive element (HRE), a cis-acting DNA sequence present upstream of several genes essential for the cellular response to hypoxia [1]. HIF-1α-responsive genes also function in the glycolysis pathway and in hematopoiesis and angiogenesis, through all of which cells acquire an hypoxia-adapted metabolism and increased oxygen supply [2]. Recently, HIF-1α has emerged as a key regulator in the growth of gastric cancer [3].

Apoptosis is an evolutionarily conserved cell death mechanism that also occurs in the adaptive cellular response to hypoxic stress. Apoptosis, too, is an important safeguard against tumor development. Tumors that exhibit loss of the p53 tumor-suppressor gene exhibit reduced levels of hypoxia-induced cell death and an associated increase in tumor progression [4]. The p21 gene (WAF1) was cloned in a genetic screen for downstream effectors of p53 and separately in a screen for upstream regulators of cyclin-dependent kinases (CDKs) as CDK-interacting protein (CIP1) [5]. The p21 promoter can be transactivated by HIF-1 in a human prostate cancer cell line, indicating that p21 is an HIF-1 target gene [6]. Furthermore, hypoxia-induced p21 expression was abrogated in cells lacking HIF-1α, but not in parental cells [7].

HIF-1α may therefore promote both cell survival and growth arrest through the induction of hypoxia-responsive genes. In the present study, we examined the role of HIF-1α in hypoxic control of tumor progression, by examining the relationship between HIF-1α expression, p21 expression and apoptosis in tissue specimens from patients with gastric cancer.

## Materials and methods

### Clinical materials

Subjects were 126 patients with gastric cancer (85 men, 41 women; age range, 27 to 88 years; mean age, 65.2 years) who underwent gastrectomy at our institution in 1994. Curative resection was performed in 77 patients and non-curative resection in 49. Resected tissue specimens were fixed in a 10% formaldehyde solution and embedded in paraffin. Sections (4 μm thickness) were mounted on glass slides. All samples were examined macroscopically and histologically, based on criteria proposed by the General Rules for the Gastric Cancer Study [8]. Histological examination was carried out on tissue preparations stained with hematoxylin and eosin (H&E). In the current study, tumors were divided into two histological types: differentiated type, comprising papillary adenocarcinoma and tubular adenocarcinoma, and undifferentiated type, comprising poorly differentiated adenocarcinoma, signet ring cell carcinoma, and mucinous adenocarcinoma. Two paraffin blocks were prepared for all patients" one containing both tumor tissue and adjacent normal tissue, and the other containing tumor tissue invading to the deepest level of the stomach wall.

### Immunohistochemical staining

All specimens were immunostained with a monoclonal antibody against p21^WAF1/CIP1 ^(SX118, diluted 1:50, DAKO, Glostrup, Denmark), p53 (Do-7, diluted 1:50, DAKO, Glostrup, Denmark), and HIF-1α (NB 100–105, diluted 1:100, Novus Biologicals, Littleton, CO, USA)[9]. After deparaffinization and rehydration, the slides for p21 and p53 immunostaining were autoclaved in citrate buffer (0.01 M, pH 6.0) at 120°C for 10 minutes; 0.001 M EDTA (pH 8.0) was used for HIF-1αimmunostaining to facilitate reactivity of the fixed embedded tissue antigen with the antibody. Endogenous peroxidase was blocked by incubating the samples in methanol containing 0.3% hydrogen peroxide for 10 minutes. Samples were then rinsed in phosphate-buffered saline (PBS) and incubated with normal rabbit serum for 30 minutes. Sections were incubated with the aforementioned primary antibodies for 2 hours at room temperature, then rinsed three times in PBS. For detection, we used a Histofine SAB-PO (M) kit (Nichirei Corp., Tokyo, Japan). The sections were then incubated with biotinylated rabbit anti-mouse immunogloblin (Ig; IgG, IgA, and IgM; Nicchirei Corp) for 10 minutes, washed three times in PBS and treated with peroxidase-conjugated streptavidin for 10 minutes. After a final washing in PBS, the peroxidase label was detected by incubating the sections in diamino-benzidine tetrahydro-chloride (DAB) for 3 minutes. Nuclear counter-staining was done using Mayer's hematoxylin solution. For negative controls, primary antibodies were replaced with non-immune, normal serum. Automated immunohistochemistry was also carried out to support the immunostaining described above, using a Ventana Discovery™ System (Ventana Medical Systems, Inc., Tucson, AZ, USA).

### Evaluation of immunostaining

**Figure 1:**
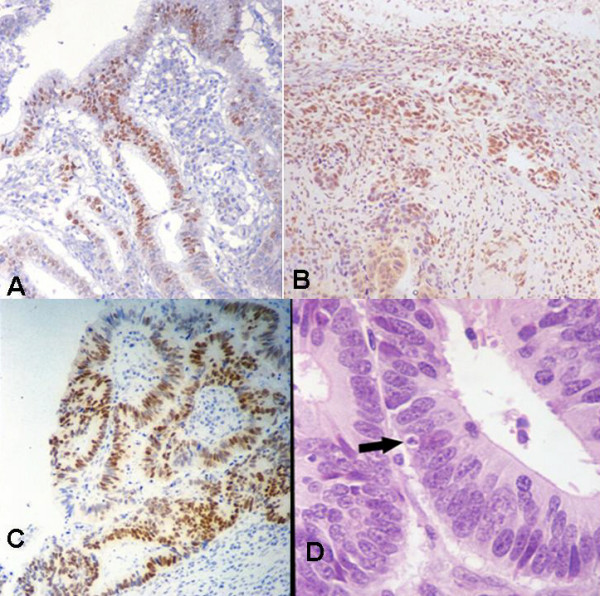
A) p21 expression in adjacent tumor tissue. Nuclear immunostaining is evident (original magnification, 100×). B) HIF-1α expression in invading regions of tumor. Variability in intensity of nuclear immunostaining is evident, accompanied by cytoplasmic staining (original magnification, 100×). C) Nuclear immunostaining of p53 in tumor tissue (original magnification, 100×). D) Tumor tissue stained with H&E (original magnification, 200×). Tumor tissue contains cells (arrow) with characteristic features of apoptosis.

p21 protein was present in the nuclei of tumor cells (Figure 1A). In some cases, normal gastric mucosa expressed p21 protein, and nuclear staining could be detected in the superficial reaches of the tumor, but not in the deeper regions. We counted the number of p21-positive cells in the whole tumor section, and evaluated the number of p21-postive cells according to the depth of layers. The pattern of HIF-1α immunostaining in the tumor was nuclear and/or cytoplasmic (Figure 1B). Nuclear staining of HIF-1α was absent in normal tissue excluding cytoplasmic staining. In all samples, p53 protein was undetectable in normal tissue, and present in the nuclei of tumor cells (Figure 1C). A cell with nuclear immunostaining for p21, p53 or HIF-1α (weak or strong) was scored as positive. Based on these criteria, areas of focal staining with the highest percentage of p53- and p21-positive nuclear staining within deep tumor tissue were estimated. A tumor was scored as p21- or p53-positive when more than 10% of the tumor cells had nuclear staining, in keeping with previous reports [10]. HIF-1α expression was frequently evident in regions around the invading edges of the tumor, and necrotic areas such as those close to a deep ulcer. We scored tumors as positive for HIF-1α overexpression if nuclear staining was detected in more than 10% of the tumor cells, irrespective of cytoplasmic reactivity at any level [9,11].

### Evaluation for apoptosis

We scored apoptotic cells as those showing the typical characteristics of apoptosis when tissue specimens were stained with H&E (Figure 1D). Apoptotic cells were identified based on the characteristic features of apoptosis: compaction and migration of nuclear and cell outlines, nuclear fragmentation and protuberance, and the presence of apoptotic bodies [12]. Apoptotic cells were counted in tissue specimens from all patients. Five high-power fields (×400) with the most abundant distribution of tumor cells were selected for counts and between 1000 and 1500 tumor cells were counted. The apoptotic index was then calculated as the percentage of apoptotic cells. Areas with extensive necrosis were avoided. A single pathologist at each institution reviewed the slides and counted apoptotic cells according to the criteria described above.

### Statistical analysis

The BMDP Statistical Package program (BMDP, Los Angeles, CA) for IBM (Armonk, NY) 3090 mainframe computers were used for all statistical analyses [13]. Data sets were compared by chi-square and Student's t-tests using the BMDP 4F and 3S programs. The BMDP 1L program was used to analyze survival time using the Kaplan-Meier method. Statistical significance was set at the P < 0.05 level.

## Results

### p21 expression and clinico-pathologic factors

Table 1 shows the correlation of the expression or loss of expression of p21 with several clinico-pathologic factors. Overall, 71 (56.3%) of the 126 tumor specimens were negative for p21 expression. In tumors from patients under 65 years of age, loss of p21 expression was significantly more frequent than in tumors from patients over the age of 65 (P = 0.026). Loss of p21 expression was significantly more frequent in tumors with large size (P = 0.03), as compared to smaller tumors. With regard to metastases, tumors with loss of p21 expression tended to show increased frequency of lymphatic invasion (P = 0.089, Table 1) and a significantly higher frequency of lymph node metastasis (P = 0.022, Table 2) than p21-expressing tumors. Table 3 shows the relationship between p21 expression, and the expression of p53 and HIF-1α. Overexpression of p53 was detected in 57 (45.2%) tumors, and HIF-1α overexpression was detected in 49 (38.9%) tumors. Immunohistochemical analysis did not reveal a correlation between p21 expression and p53 expression, or p21 and HIF-1α overexpression (P = 0.444 and 0.609, respectively).

**Table 1:** Relationship between p21 expression and clinico-pathologic factors

Factors	p21(+) (n = 55)	p21(-) (n = 71)	P value
Age (years)			
<65	20	40	**0.026**
65≦	35	31	
Gender			
Male	38	47	0.731
Female	17	24	
Depth of invasion			
t1	33	35	0.231
t2,3,4	22	36	
Histology			
Differentiated	35	39	0.325
Undifferentiated	20	32	
Tumor size			
<3 cm	23	22	**0.03**
3 cm≦	22	49	
Lymphatic invasion			
Negative	26	23	0.089
Positive	29	48	
Venous invasion			
Negative	42	57	0.595
Positive	13	14	

**Table 2:** Relationship between p21 expression and metastasis

	p21-negative tumor (%)	P value
Lymph node metastasis		
Negative	36/75(48.0)	**0.022**
Positive	35/51(68.6)	
Liver metastasis		
Negative	68/122(55.7)	0.447
Positive	3/4(75.0)	
Peritoneal dissemination		
Negative	62/112(55.4)	0.525
Positive	9/14(64.3)	

**Table 3:** Relationship between p21 and p53, and HIF-1α expression

		p21		
			
		Positive (n = 55)	Negative (n = 71)	P value
p53	Positive (n = 57)	27	30	0.444
	Negative(n = 69)	28	41	

HIF-1α	Positive (n = 49)	20	29	0.608
	Negative(n = 77)	35	42	

### Apoptosis associated with HIF-1α and p21 expression

The mean apoptotic index of all 126 tumors was 8.95 ± 6.24 (range 0–37). Tumors were divided into four different populations based on HIF-1α and p21 expression and the mean apoptotic index for each group was calculated (Table 4). Tumors that were HIF-1α-positive/p21-negative had the lowest apoptotic index. There was a significant difference between tumors that were HIF-1α-positive/p21-negative, and those that were HIF-1α-negative/p21-negative (P = 0.037).

**Table 4:** Apoptosis associated with HIF-1α and p21 expression

Factor	Apoptotic index
HIF-1α (-), p21(+) n = 35	9.6 ± 7.33***
HIF-1α (-), p21(-) n = 42	9.83 ± 6.96**,***
HIF-1α (+), p21(+) n = 20	8.85 ± 4.04*,**,***
HIF-1α (+), p21(-) n = 29	6.97 ± 4.22*,**,***

* P = 0.129 ** P = 0.037 *** P = 0.078 (mean ± S.D.)

### Clinical outcome associated with HIF-1α and p21 expression

The mean follow-up time for patients was 55 ± 28 (± 1 S.D.) months (range, 1–82 months). The 5-year survival rate of patients with p21-negative tumors was lower than that of p21-positive tumors, but the difference was not statistically significant (data not shown). The 126 patients were again divided into four populations based on HIF-1α expression and p21 expression, and we examined the relationship between HIF-1α expression and prognosis in p21-positive or -negative tumor samples. Table 5 shows the 1-, 3- and 5-year survival rates for patients and the correlation with HIF-1α and p21 expression. Patients with HIF-1α-positive/p21-negative tumors had a significantly poorer prognosis than the other study populations. In particular, in patients with HIF-1α-positive tumors, those who had lost expression of p21 had a significantly poorer prognosis than those with p21 expression (P = 0.042).

**Table 5:** Year survival rate associated with HIF-1α and p21 expression

Factor	1-year survival	3-year survival	5-year survival
HIF-1αg(-), p21(+) n = 35	94.1	82.0	82.0***
HIF-1α (-), p21(-) n = 42	92.7	80.0	80.0**,***
HIF-1α (+), p21(+) n = 20	95.0	85.0	70.0*,**,***
HIF-1α (+), p21(-) n = 29	68.1	57.4	45.9*,**,***

* P = 0.042 ** P = 0.003 *** P = 0.003 (%)

## Discussion

Our findings show that loss of p21 expression correlated positively with younger patient age, and larger tumor. Moreover, many patients with p21-negative tumors had lymph node metastasis when compared to those with p21-positive tumors, at a significantly higher frequency. These results suggest that the loss of expression of p21 is involved in the processes of tumor growth and metastasis, in agreement with previously reports [14,15].

HIF-1α overexpression has been linked to a poor clinical outcome in some types of human cancers [16-18]. However, some reports have suggested that tumor expression of HIF-1α does not confer a survival advantage [18,19]. Although most of the HIF-1 target genes can promote tumor growth through their enhanced expression, HIF-1-activated genes, including p21, also have the potential to inhibit growth under hypoxic conditions [20,21]. Ectopic expression of HIF-1α in endothelial cells resulted in up-regulation of p21, reduction of CDK activities, cell cycle arrest at the G_0_/G_1 _check point, and subsequent apoptosis [22]. In the current study, we found that apoptotic cells were under-represented in HIF-1α-positive/p21-negative tumors. Under hypoxic conditions, HIF-1α may inhibit tumor proliferation through p21-mediated cell cycle control, resulting in the selection of cells that are resistant to apoptosis and anti-cancer treatments. Most tumor cells retain the ability to undergo apoptosis in response to hypoxic stress [23]. When the apoptotic response to hypoxia is lost, emerging tumor cells may be more resistant to treatment and may therefore contribute to subsequent tumor relapse [24]. The mechanisms by which hypoxia selects for cells resistant to apoptosis is unclear, but the involvement of the p53 mutation has been examined [25]. Reports have shown that hypoxia inhibits cell growth, and may cause apoptosis through a p53-dependent pathway [26]. HIF-1α has also been shown to promote p53-dependent apoptosis [27], but other studies have shown that growth arrest in response to hypoxia is p53-independent [26]. In the current study, we found no evidence of a relationship between p53 and p21 expression. We also evaluated the relationship between HIF-1α and p53 expression to cell apoptosis, but found no statistical significance between HIF-1α and p53 expression (data not shown). Our previous study showed that the combination of HIF-1α overexpression with nonfunctional p53 tended to indicate a dismal prognosis [28].

In patients with HIF-1α-positive tumors, the correlation between loss of p21 expression and poor clinical outcome may reflect a physiological difference in the ability of p21-positive versus p21-negative tumors to survive under hypoxic conditions. Although HIF-1α-dependent transcriptional activation has been associated with tumor growth, our results suggest that concomitant expression of p21 and HIF-1α may retard tumor growth to some degree.

The molecular mechanism underlying HIF-1α expression in cancer warrants particular attention [29]. The widespread occurrence of upregulated HIF-1α in common cancers and the involvement of hypoxia pathways in tumor angiogenesis certainly argue for its importance and wide applicability. Chemotherapy and radiation that target HIF-1α may be effective and realistic, and in fact, this approach has been reported [30]. However, the qualitative and quantitative differences in the hypoxic response of different cell types are not well known [31]. Further research is therefore required in order to evaluate the effects of HIF-1-mediated pathways on cell proliferation and apoptosis in human cancers under hypoxic microenvironments.

In the present study, we showed that HIF-1α overexpression and loss of p21expression in gastric cancers correlated with poor patient prognosis, compared to tumors that retained p21 expression, or had lost HIF-1α expression. A potential mechanism for this was suggested by the finding that apoptotic cells were under-represented in HIF-1α-positive/p21-negative tumors. Aggressive tumors that fail to induce p21 in an HIF-1α – dependent manner may have increased cell survival without apoptosis, and contribute to a poor prognosis for patients.

## Conflict of interest

The author(s) declare that they have no competing interests.

## Authors' contributions

**KM **carried out the immunohistochemical study and performed the statistical analysis, and drafted the manuscript.

**YK **conceived the study, and participated in its design and coordination and helped to draft the manuscript.

**SO **involved in drafting the manuscript and revising it critically for important intellectual content.

**YM **gave final approval of the version to be published.

All authors read and approved the final manuscript.

## Funding support

Department of Surgery and Science, Graduate School of Medical Science, Kyushu University, Fukuoka, Japan.
